# Are attributes in “true” diagnostic classification modelling studies theoretically distinct from those in other studies?

**DOI:** 10.3389/fpsyg.2026.1932069

**Published:** 2026-10-13

**Authors:** Adam Coates

**Affiliations:** Department of Education, University of Bath, Bath, United Kingdom

**Keywords:** cognitive diagnostic assessment, diagnostic classification model, latent class model, philosophy of measurement, realism

## Abstract

Diagnostic classification modelling (DCM) encompasses both a family of latent class models and a specific methodology for using these models with an assessment. Critics argue that research employing the latent models without the methodology should not be labelled DCM. However, most DCM studies fall into this category. This paper evaluates the legitimacy of these studies by testing whether a theoretical boundary exists between the attributes used in cognitive models in “true” and “other” DCM research. This is important because “other DCM” studies often suffer from model fit issues, and efforts to address this rely on the theoretical framework of “true DCM.” Theoretical distinctions may arise because “True DCM” typically use fine-grained attributes representing procedural steps, whereas “other DCM” often use broad attributes which refer to curriculum topics. Three intuitive boundaries are evaluated: singular attributes versus composites of finer-grained skills; attributes as real processes versus attributes as constructs; and attributes representing individual-level problem-solving versus group-level behaviour. The paper argues that none of these distinctions hold, suggesting that both “other” and “true DCM” can share the same core theoretical assumptions across these three aspects. However, this should not be interpreted as giving researchers carte blanche; other theoretical distinctions may well exist, and the use of broad attributes can carry notable practical challenges. Instead, DCMs with broad attributes may be best suited for a narrow range of assessment contexts.

## 1 Introduction

“Diagnostic classification modelling” (DCM) can refer to the cognitive diagnostic assessment (CDA) methodology for producing diagnostic scores (Leighton and Gierl, 2007b). The starting point of the methodology is a cognitive model. This is a simplified representation of problem solving, and includes attributes - the knowledge and cognitive processes required for completing the task (Gierl et al., 2007; Leighton and Gierl, 2007a). Cognitive models are typically designed based on task analysis, think-aloud protocols, curriculum structure, and/or theory (Gorin, 2006; Ketabi et al., 2021; Leighton and Gierl, 2007a). For an example, Table 1 presents the attributes in a cognitive model for learning probability in middle school mathematics (Bai, 2020).

**Table 1:** Attributes in the cognitive model of learning mathematical probability

Attributes
A1: Randomness: distinguish between certain events, random events, and impossible events.A2: One-dimensional sample space: list all possible outcomes of a one-dimensional event.A3: Two-dimensional sample space: list all possible outcomes of a two-dimensional event.A4: One-dimensional probability comparisons: compare the probability of one-dimensional events.A5: Two-dimensional probability comparisons: compare the probability of two-dimensional events.A6: Probability of a one-dimensional event: calculate the probability of a one-dimensional event by definition.A7: Probability of a two-dimensional event: calculate the probability of a two-dimensional event by definition.A8: Probability estimation of a one-dimensional event: estimate the probability of a one-dimensional event by frequency.A9: Probability estimation of a two-dimensional event: estimate the probability of a two-dimensional event by frequency.

*Note:* From Bai (2020, p. 3).

In the CDA methodology, after a cognitive model has been established, an assessment is created based on the model. This involves designing test items so that there is sufficient coverage of the attributes in the model (Lin and Xu, 2024). Then, responses to the assessment are collected and these are analysed using a latent class model. The results of the model can be used to diagnose which attributes each student has mastered and where they may need support (Chen et al., 2010; Sessoms and Henson, 2018). For example, if a student gives an incorrect answer for writing the number of possible answers when randomly selecting a number from the set {1, 2, 3, 4, 5} (Bai, 2020, p. 5), this can indicate they have yet to master A1 (*randomness*) and/or A2 (*one-dimensional sample space*).

“Diagnostic classification modelling” can also refer specifically to the latent class models that are used within the CDA methodology (Rupp and Templin, 2008). For example, the Handbook of Diagnostic Classification Models (Von Davier and Lee, 2019) is focused almost exclusively on statistical topics, and contains few references to creating cognitive models or interpreting results. This has led to a distinction between what some call “true DCM” which follow the CDA methodology (Gierl and Cui, 2008; Ravand and Baghaei, 2020) and, what we may coin, “other DCM” studies which use the statistical models but not the methodology. Reviews of DCM studies show that “other DCM” studies actually outnumber “true” studies (e.g., Ravand and Baghaei, 2020; Sessoms and Henson, 2018).

A critical area in which “true DCM” studies are often quite different to “other DCM” studies is the specification of attributes. In the CDA methodology, attributes are generally fine-grained and refer to clear stages in a solution (e.g., *separating a whole number from a fraction*: Tatsuoka, 1990), while in studies using DCM methods only, attributes are typically broader and may explicitly contain multiple skills (e.g., *reading, interpreting, and representing data*: Zhu, 2023).

This paper seeks to establish whether a theoretical distinction can be made between attributes typical for “true DCM” and those typical for “other DCM.” If the attributes are different in type, then an important distinction can be made between “true” and “other” DCM. As a result, an alternative conceptual and methodological framework is required for “other” studies because they are engaging in a fundamentally different activity (Gierl and Cui, 2008). However, if the attributes are superficially distinct and differ only in degree, then it is arguable that the framework of DCM can be used equally in both cases. While this may seem trivial, it can have important practical consequences because “other DCM” studies currently mimic the “true DCM” methodology. This can result in inappropriate methods being used, such as omitting items when they have poor fit statistics, rather than adapting the cognitive model to fit the items.

### 1.1 The retrofitting debate

The most prevalent form of “other DCM” occurs through the practice of retrofitting. This approach deviates from the CDA methodology by designing a cognitive model *after* an assessment has been conducted. While this divergence means that these studies are not considered “true DCM,” systematic reviews have found that retrofitting studies are more common than “true” studies (Ravand and Baghaei, 2020; Sessoms and Henson, 2018).

There is significant debate in the DCM community about the value of retrofitting studies. Multiple layers of criticism have been levelled against the practice: (A) retrofitting often occurs with assessments designed to be unidimensional, and therefore may not be able to provide valid multidimensional scores; (B) because the test items are fixed, it may not be possible to create a cognitive model that comprehensively captures the skills required in a test while having sufficient items per attribute; (C) the two previous issues result in compromises that are likely to produce poor model fit; (D) it may not be possible to create a cognitive model that is useful for helping teachers understand students’ thinking; and (E) retrofitting studies rarely make sufficient effort to validate their cognitive models (Bradshaw et al., 2014; Gierl et al., 2010; Gierl and Cui, 2008; Henson, 2009; Jurich and Bradshaw, 2014; Sessoms and Henson, 2018). It is for these reasons that Gierl and Cui (2008) make the argument that retrofitting studies should not be included in the “diagnostic classification modelling” label.

On the other side of the debate, authors of retrofitting studies often acknowledge these limitations, accepting that the practice can compromise the theoretical validity, diagnostic validity, and practical utility of the results. Nonetheless, they argue that even if the results of retrofitting may be suboptimal, they can nonetheless provide useful feedback on student learning (e.g., Chen and Torre, 2014; Lee and Luna-Bazaldua, 2019). A notable contribution in this regard comes from Liu et al. (2018) who provide a guide for retrofitting DCM. They outline four iterative stages to produce the best possible DCM model using data that has already been collected, and identify key methods that can improve model fit (e.g., if an attribute is designed such that it is the only skill tested in some items, it will have higher classification accuracy; Liu et al., 2018).

Retrofit studies often find poor fit between the data and the DCM, and this gives a further reason why it can be important to determine whether there is a theoretical boundary between “true” and “other” studies: if “other” studies are theoretically distinct, then efforts to improve model fit may be misguided, since the DCM methodology and statistical model may be unsuitable for the context.

### 1.2 Retrofitting and apparent differences in attributes

The practice of retrofitting is important because common practices in retrofitting illustrate clearly how attributes in “other DCM” studies often appear different from those in “true DCM” studies. First, many retrofitting studies seek to analyse large-scale datasets, but these tend to be holistic assessments of a school-subject (e.g., mathematics, science, or reading). In these cases, attributes are necessarily broad in scope to match the broad scope of the assessment. For example, retrofitting studies of the Programme for International Student Assessment (PISA) use *quantity* and *uncertainty and data* as attributes (e.g., Liang et al., 2022; Wu et al., 2020). In contrast, “true” studies within the CDA methodology tend to focus on specific topics within a subject, meaning that attributes are fine-grained. For example, Attali and Arieli-Attali (2019) designed an assessment focusing on rational number operations and had attributes such as the *concept of half* and *fraction as a single number*, and Zhan et al. (2023) analysed an assessment testing line symmetry, including the attributes *understanding the concept of equilateral triangles* and *drawing perpendicular bisectors*.

A complementary feature common to retrofitting studies is the use of explicitly compound attributes. When retrofitting, it is not uncommon to face the challenge that two concepts are always measured together or that some concepts are measured by very few items. In such cases, researchers often combine related concepts into a single attribute (R. Liu et al., 2018). As a result, it is easy to find compound attributes in retrofitting studies, such as *measure, estimate, and understand properties of lines and angles* (Lee et al., 2011) and *identify properties of figures by observing, measuring, or drawing* (Chen et al., 2019). In “true DCM,” explicitly compound attributes are less common because the assessment is designed after the cognitive model, meaning that sufficient items can be created to distinguish between attributes.

The context of retrofitting illustrates why a DCM study may use broad and compound attributes; however, this practice is not limited to retrofitting alone. Even when assessments are designed based on cognitive models, studies can be found with such attributes. For example, Chen et al. (2017) created a cognitive model and assessment for the number sense of primary school students, and include the compound attribute *operations with fractions and decimals*; and Li et al. (2016) used a longitudinal DCM to analyse a holistic mathematics assessment, and their cognitive model included broad attributes such as *measurement* and *problem* solving. While these studies may not face some of the model fit issues that can occur when retrofitting, they nonetheless need to address the challenge that their cognitive models may not be suitable for a DCM.

## 2 Research focus

At first glance, whether the attributes in the cognitive models of “other DCM” are of the same theoretical type as those of “true DCM” may seem inconsequential. Regardless of the order in which a cognitive model and assessment are created, poor model fit is poor model fit, and if the attributes used in an “other DCM” are not suitable for the concept of a “cognitive model,” we may simply use alternative terminology, e.g., an “attribute model.”

However, the issue arguably runs deeper than terminology. If we take the realist position that underpins cognitive diagnostic assessment (e.g., Leighton and Gierl, 2007b; Rupp and Mislevy, 2007), then the cognitive model and DCM are reflections of student cognition. If attributes in “true” and “other DCM” studies are theoretically distinct, a misalignment between reality and the diagnostic model is the likely cause of the model fit issues that often occur in “other” studies. This is important because, at present, “other DCM” piggyback on the methodology of “true DCM,” making adjustments where deemed necessary (e.g., as in: Lee and Luna-Bazaldua, 2019; Liu et al., 2018). This approach is likely to be ineffective if attributes used in “other” studies are fundamentally distinct from those in “true” studies. For example, it can be inappropriate to use think-aloud protocols to validate broad attributes, since a student is unlikely to express that they are using *measurement* or *algebra* skills to solve a task. Therefore, the nature of attributes in “other DCM” is important to establish whether a completely distinct methodology is required, or whether it is suitable to adopt and adapt methods from the cognitive diagnostic framework.

There are three intuitive and interconnected arguments that could be used to establish a theoretical distinction between the attributes common to “true DCM” and “other DCM.” These distinctions are proposed by the current author, since this topic, and the philosophical foundations of DCM in general, receive limited attention in the literature (Norris et al., 2007):

1. the attributes used in “true” models are singular and indivisible, whereas the attributes in “other” models are composites of finer-grained skills and knowledge;
2. the attributes in “true” models refer to ontologically real processes during problem solving, whereas the attributes in “other” models are ontological constructs;
3. “true” models represent how problems are solved at the individual-level, whereas “other” models can only represent problem-solving at the group-level.

The theoretical validity of each of these potential distinctions will now be explored in turn. To provide an anchor for the discussion, the broad-grain attribute *algebra* will be contrasted with the finer-grained attributes in the paradigmatic cognitive model of fraction subtraction, originating from the work of Tatsuoka (1984, 1990). The use of *algebra* as an attribute is common in DCM studies of international large-scale assessments, such as the model used by Gu (2023) using the assessment framework of TIMSS 2019 (Table 2). The fraction subtraction dataset from Klein et al. (1981) and Tatsuoka (1984) is perhaps the most analysed data in the field of DCM, and the attributes for solving subtraction tasks by separating mixed numbers into whole and fraction parts are shown in the right-side column of Table 2. Examples of how these attributes map to task solutions are given later in a figure.

**Table 2:** Attributes in the cognitive models of TIMSS and of fraction subtraction

Attributes in the cognitive model of Gu (2023)	Attributes in the cognitive model of Tatsuoka (1990)
Number	Convert a whole number to a fraction
Algebra	Convert first mixed number to fraction
Geometry	Convert second mixed number to fraction
Data and probability	Simplify before subtracting
Knowing	Find a common denominator
Applying	Column borrow to subtract numerator
Reasoning	Reduce answer to simplest form

It is important to concede that these examples differ in more ways than just granularity. First, *algebra* is an attribute in an assessment of the full mathematics domain, while the fraction subtraction assessment is much narrower in scope. Second, international large-scale assessments aim to evaluate country-level learning states, while the fraction subtraction assessment diagnoses individual students. Additionally, the assessment framework for TIMSS, for example, is created to align with curricula, whereas the fraction subtraction model was informed by observation of student responses. As a result, differences between the models may not stem solely from the consequences of different attribute sizes, but reflect the broader contexts in which the cognitive models are typically applied.

### 2.1 Attributes as composites and attributes as parameters

The CDA literature can be a little unclear regarding the nature of attributes. There is general agreement that attributes are knowledge structures, cognitive processes, and skills required for solving a particular task (Chipman et al., 1995; Gierl et al., 2007; Gierl and Cui, 2008; Leighton and Gierl, 2007b; Rupp and Mislevy, 2007). There is also agreement that attributes should be fine-grained (Gierl and Leighton, 2007; Rupp and Templin, 2008), but there are different perspectives as to what this means.

As discussed, one fundamental difference between attributes in “true” models and those of “other” models is the level of granularity. This can suggest a conceptual difference between the structure of attributes in the two approaches: fine-grained attributes refer to single processes, whereas broad attributes are collections of finer-grained processes. This conceptualization may have intuitive appeal, as it is easy to see how *converting a whole number to a fraction* may be considered a single step in a solution, while *algebra* contains many processes and skills. Borsboom and Mellenbergh (2007) classify this distinction as “attributes as parameters” and “attributes as composites.”

This distinction has important consequences for the relationship between the cognitive model and DCM. In the DCM, attributes are generally represented as binary. If fine-grained attributes are singular, there is clear alignment with the representation in the DCM. In contrast, if broad attributes are composites, then the all-or-nothing representation is potentially a poor representation of student learning.

Despite the face-value appeal of this distinction, arguing that “true” cognitive models contain attributes as parameters is challenging. Cases where an attribute can truly be considered singular are rare, and even seemingly unitary attributes such as *converting a whole number to a fraction* (Tatsuoka, 1984) can contain multiple processes. Consider that converting the whole number involves two distinct stages: *identifying the target denominator* and *multiplying the whole number by that intended denominator*. In the fraction subtraction context, *identifying the target denominator* could be considered to follow a single process because the items are very simple. However, the multiplication stage could follow various methods, using rote memory or by repeated addition, for example.

In fact, there is general agreement in the CDA literature that attributes are typically composites. Mislevy (2018) describes attributes as clusters of lower-level knowledge and skills. Elsewhere, there is recognition that attributes can exist at multiple levels of grain-size, meaning that it is valid to have cognitive models with attributes that can be divided into finer-grained processes and skills (Gierl and Leighton, 2007). In fact, Leighton and Gierl (2007a) criterion for attribute grain size is pragmatic rather than conceptual: attributes should be sufficiently precise for providing useful feedback, rather than being the finest-grain possible. In the same vein, Chipman et al. (1995) note that useful (i.e., simple) cognitive models are often composed of broad attributes.

The preliminary conclusion is that it may be possible to design cognitive models with attributes as parameters, but the use of composite attributes is common. As a result, a theoretical distinction between “true” models and “other” models cannot be established by classifying the former as using attributes as parameters and the latter as using attributes as composites.

However, this may be considered too simplistic. Although something may be composed of smaller units, it can nonetheless be considered single. A human being is composed of cells, but that does not mean a person does not function as a single unit. Boyd (1991), for example, argues that a composite of smaller parts can be considered single if the parts are reliably linked and kept together by sustaining mechanisms. This means the cognitive process of *converting a whole number to a fraction* can be both a composite and singular because the sub-processes combine into a fixed routine (Guyon et al., 2017).

At first glance, *algebra* appears too disparate to be a fixed routine: solving a linear equation and factoring a quadratic equation follow distinct processes. However, if the first steps in the *algebra* routine are identifying the task as an algebra problem, selecting an appropriate solution sub-routine, and coordinating the employment of algebraic subskills, then *algebra* can indeed be considered a single routine despite containing diverse sub-processes. This is because the executive coordination of algebra skills serves as the sustaining mechanism.

It can be argued, therefore, that although attributes in “true” and “other DCM” typically differ in granularity, grain-size is not a deciding factor in whether an attribute is theoretically single or composite. Instead, the real issue is whether the attribute can be treated as a sufficiently stable whole, and while this may occur more often with finer-grained attributes, broad attributes can still be considered single. As a result, the attributes of “true DCM” and “other DCM” are not necessarily distinct in this regard.

### 2.2 Real and constructed attributes

While attributes in both “true DCM” and “other DCM” may be composites of finer-grained skills and processes, an alternative ontological distinction could be raised. Within the CDA framework, cognitive models have been described explicitly as intended to model real cognition and behaviours (Corter, 1995; Rupp and Templin, 2008). If the broad attributes in “other DCM” can only be considered ontological constructs rather than real, this would be a critical difference between the two DCM approaches. Again, this distinction can be intuitively attractive. It seems reasonable to say that *converting a whole number to a fraction* is a specific underlying skill for solving fraction equations, as in Figure 1. In contrast, *algebra* can be seen as a label to describe a collection of associated mathematics tasks (i.e., a construction).

This is critical because the ontological nature of an attribute is determined by the relationship between the attribute and its indicators. For a realist relationship, the attribute underlies the indicators; we might say, for example, that a person smiles (indicator) because they are happy (latent variable). For the constructivist relationship, the attribute is a kind of summary of the indicators; we might say that happiness is what we label a set of distinct behaviours which typically occur together, including smiling, making eye contact, and being animated.^1^

Under this framework, *converting a whole number to fraction* can be understood to represent actual cognitive processes that students engage in, even if these processes differ across students. In this case, it is the ontologically real cognitive process of converting a whole number which results in a correct response to an item. Conversely, *algebra* can be conceived as representing a social category which includes a range of distinct tasks with no single underlying mechanism, i.e., interpreting graphs, identifying sequences, and rearranging equations may be real cognitive processes, but *algebra* is not. This means *algebra* may be understood a label used to structure curricula rather a representation of a specific type of cognition – a student does not solve a task correctly because they “did algebra.*”* In this case, an attribute is no more than the sum of its parts, as reflected in the direction of the arrows in Figure 1.

**Figure 1:**
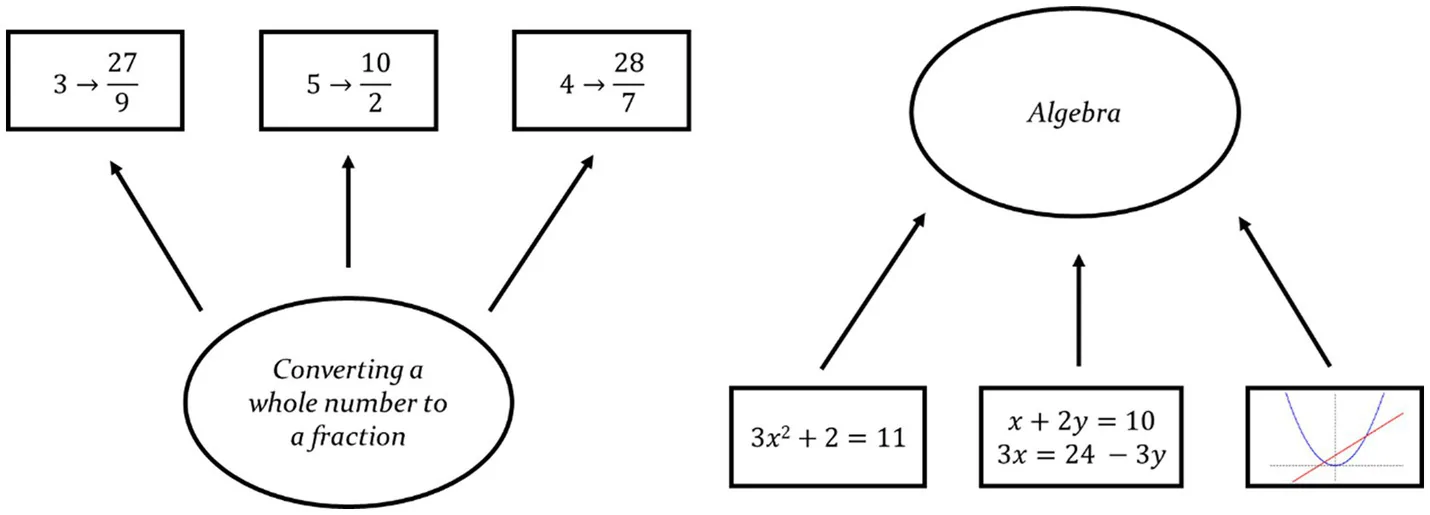
A realist model for converting a whole number to a fraction and constructionist model for algebra.

Crucially, the different structures between attributes and indicators are reflected in statistical models. If ontologically real attributes are assumed, a reflective model is used (Edwards and Bagozzi, 2000). In this case, a regression of the indicators on the latent variable is assumed to fully explain the covariation between indicators. This is because the latent variable is assumed to be the sole common cause of the indicators (Hood, 2013). As a consequence, the indicators are expected to vary independently after conditioning on the latent variable, i.e., they have local independence (*e* in Figure 2). In contrast, if attributes are assumed to be constructs, a formative model is used (Borsboom, 2005). In this case, the attribute is regressed on the indicators, and shared variance among subsets of indicators is possible, i.e., collinearity (*s* in Figure 2). This is because the indicators are not the result of a single common cause (the attribute). Without a single underlying cause to account for their shared variance, direct relationships or overlaps between individual indicators naturally remain (Bollen and Lennox, 1991).

**Figure 2:**
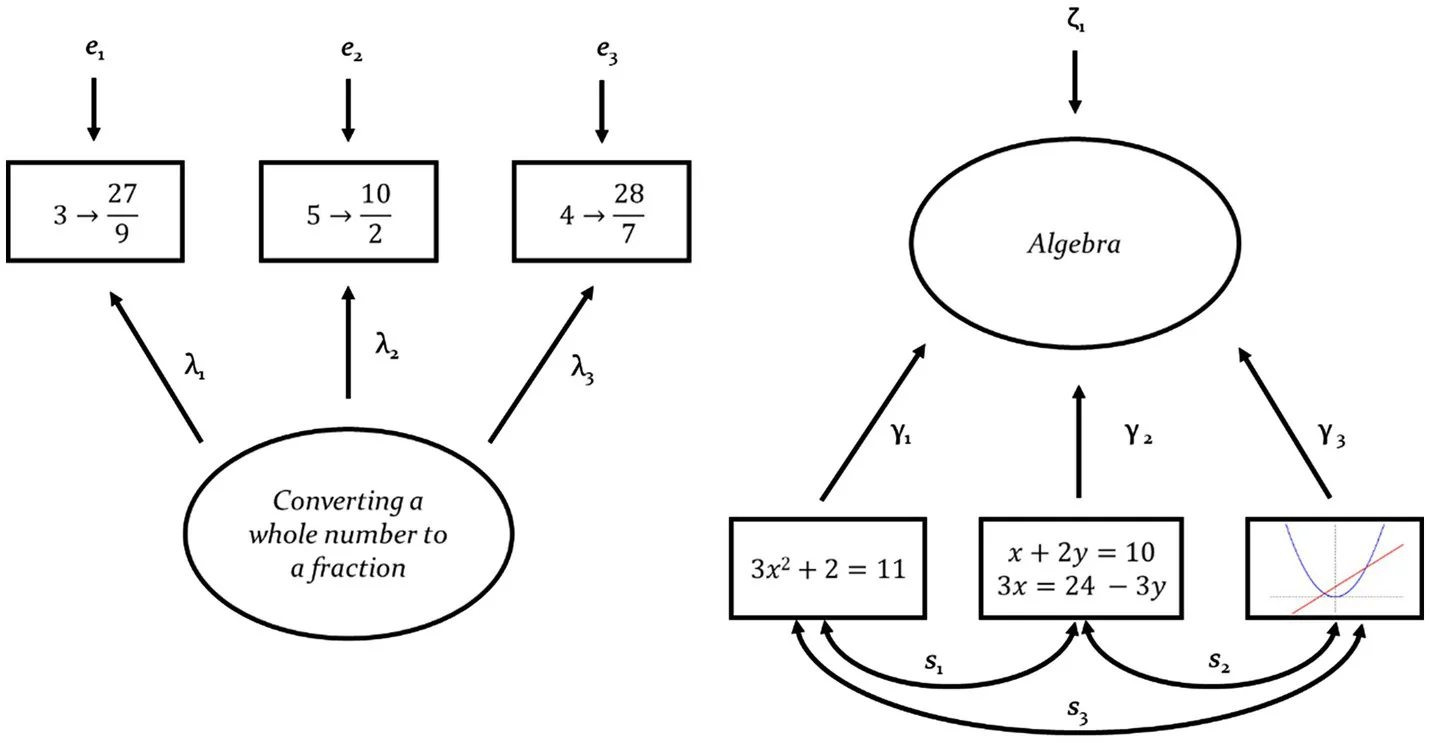
A reflective model for *converting a whole number to a fraction* and formative model for *algebra*. In this figure, e are errors, λ are factor loadings, γ are weights, ζ is a residual, and s are covariances.

As latent classification models, the item response functions of DCM represent the realist assumption. For illustration, Equation 1 presents the item response function for the DINA DCM with two attributes (de la Torre, 2009).

$$P(X_{nj}=1\alpha_{n})={\left(1-s_{j}\right)}^{\eta_{nj}}\cdot g_{j}^{1-\eta_{nj}}$$ (1)

The equation represents the probability that a respondent with attribute mastery profile $\alpha_{n}$ will answer item $X_{j}$ correctly. Here, $s_{j}$ is the *slip* parameter representing the probability that a respondent who has mastered all necessary attributes for item j nonetheless answers incorrectly; $g_{j}$ is the *guess* parameter representing the probability that a respondent who has not mastered every necessary attribute answers correctly; and $\eta_{nj}$ is 1 if all necessary attributes are mastered and 0 otherwise ( $\eta_{nj}=\prod_{k=1}^{2}\alpha_{nk}^{q_{nk}}$ , with $q_{nk}\in \{0,1\}$ depending on whether item j requires attribute k ).

The DINA response function demonstrates that item responses depend only on attribute mastery (via the $\eta_{nj}$ ) and the guessing and slip parameters of the item ( $s_{j},g_{j}$ ). It therefore is a reflective model because item responses are driven solely by students’ mastery and item parameters. Moreover, the same relationship is found in more general DCMs, such as the GDINA (De La Torre, 2011) and GDM (Von Davier, 2019), implying the same adoption of a reflective model.

In a small selection of models, deviations from this assumption can be found, such as the joint testlet model of Zhan et al. (2018) which addresses the situation where items sharing the same stimulus can be expected to have shared variance. However, these deviations are special cases of local dependence rather than the general collinearity expected for an ontological construct, i.e., by including the specific local dependencies in the model, local independence is restored.

Now, this argument does not establish whether broad attributes are ontologically real or constructs; instead, it establishes that DCMs structurally represent attributes as though they are real. Therefore, it is possible that researchers exclusively follow an instrumental approach, adopting the DCM framework for the utility of its results, rather than the theoretical consistency of its structure. Two examples from international large-scale assessments indicate that this is not always the case.

First, much like approaches in the CDA framework (e.g., Bradshaw et al., 2014; Keehner et al., 2016), item design and validation in large-scale assessments often involve cognitive interviews to determine whether respondents are drawing on broad attributes such as *algebra* (Leighton, 2012). This implies that the abilities are assumed to be real since they exist apart from, and prior to the measurement. Second, large-scale assessments often use a rotated booklet design, meaning that each student only receives a subset of items (e.g., Aldrich et al., 2024). This implies an assumption that indicators are interchangeable in the measurement of attributes because the attribute mastery of different students is estimated by their responses to different items. This is reasonable if attributes are ontologically real and the underlying cause of item responses because the mastery of attributes is independent of the specific items testing that mastery. On the other hand, if attributes are considered to be ontological constructs, items cannot be switched since attribute mastery is nothing more than a composite of the responses to a specific set of items. These two examples demonstrate broad attributes are treated as though they are ontologically real, in addition to being modelled as though they are real.

Taken together, this suggests that broad attributes are considered to be ontologically real in some cases; however, this does not establish whether making this assumption is valid. But this issue has already been addressed: In a formative model, the causes of item responses are disparate – each indicator may have a separate, distinct cause, or there may be a family resemblance situation where groups of indicators share a cause, but no single cause underlies all indicators. In the reflective model, the cause of all item responses is single — a condition that a broad attribute can meet if it is functionally single, as shown to be possible in the previous section.

In summary, grain-size is not a deciding factor in whether an attribute is assumed to be real or a construct. As a result, there is not a theoretical distinction between broad and fine-grained attributes in this regard.

### 2.3 Stochastic subjects and subpopulations

A final effort to establish a theoretical distinction may be made by arguing that the cognitive models in “true DCM” represent specific steps in problem-solving, whereas cognitive models in “other DCM” are imprecise, so only represent solving a problem as a whole. This can create a distinction because it implies that in “true DCM,” the item response function can represent an individual’s likelihood of answering an item correctly, whereas in “other DCM,” the function can only represent a sampling likelihood.

As indicated in the previous section, both “true” and “other” DCM assume a real relationship between attribute mastery and item responses. This relationship is often interpreted as causal: mastery of relevant attributes causes a respondent to solve a problem correctly. This may seem relatively uncontroversial, but there are two distinct ways in which the causal relationship can be interpreted, and these bring important implications.

For “other DCM,” broad attributes such as *algebra* and *whole number operations* may be conceptualised in the same way as a subject score such as *mathematics*. In these cases, attributes are understood as the common cause of the item response (Borsboom, 2005). A common cause refers to the situation where phenomenon A has a causal effect on B and C, but B does not have a causal effect on C (or v.v.); for example, the habit of smoking is both the cause of carrying a lighter and developing lung cancer, but carrying a lighter does not cause lung cancer (Hernán and Robins, 2020). This means that mastery of the *algebra* attribute is considered the underlying cause of success in different algebra tasks, but the processes involved in these solutions may differ. For example, solving a linear equation 4x+11=47 and simplifying exponents $2x^{5}*3x^{2}$ require different approaches, but these approaches both draw from *algebra* ability (or, in a different model, from *mathematics* ability).

For “true” models, the fine-grained attributes are intended to describe the specific processes and knowledge required to solve a problem, rather than a general set of skills (Gierl et al., 2007; Leighton and Gierl, 2007a). While we have noted that these attributes are often composites of even finer skills, the argument can be made that the process of *converting a whole number to a fraction* can be considered functionally the same for each respondent. As a result, we may expect the same process would be used by one respondent across multiple instances. In this case, mastery of the attribute can be considered a direct cause because it describes the same process across all instances of solving a type of problem, much like the habit of smoking can be the direct cause of carrying a lighter yesterday, today, and tomorrow.

The nature of the causal relationship is important because the probability of being correct in a response function can be interpreted in two ways: as an individual’s true score (the stochastic interpretation) or as the mean response probability of a subpopulation of individuals who share the same attribute mastery (the sampling interpretation). In the stochastic interpretation, the probability represents the likelihood that a specific individual with attribute profile $\alpha_{n}$ answers the item correctly; in the subpopulation interpretation, the probability represents the likelihood that a randomly chosen individual from a sample of individuals with attribute profile $\alpha_{n}$ answers the question correctly (Borsboom, 2005).

For the stochastic interpretation, we need the property of local homogeneity – that the item response function for multiple responses of a single individual is identical to the item response function for single responses of multiple individuals (Ellis and Van Den Wollenberg, 1993). This is arguable if the cognitive model sufficiently describes how solutions are reached, meaning that we can say if both Jane and James are masters of *converting a whole number to a fraction,* they will solve a problem following the same process. Moreover, if John, a non-master of converting *a whole number to a fraction*, were a master like Jane and James, he would solve the problem following the same process as Jane and James.

For the attributes typical of “other DCM,” it is clear that local homogeneity is not a valid assumption. For example, masters of *algebra* may use different response processes when finding the value of x if $3x^{2}+11=2$ . This is not a problem and simply requires the subpopulation interpretation is adopted.

For “true” models, it could be argued that the specificity of attributes is sufficient to describe response processes such that two individuals with the same mastery can be expected to follow the same process. While it seems unlikely that the exact same process may be used in each stage in a solution, we might assume the variations in processes are negligible at this fine-level of analysis. For example, converting 3 into $\frac{27}{9}$ involves basic multiplication. As mentioned earlier, it is possible that producing the numerator may come from rote or repeated addition. If we can assume that the multiplication strategy has no meaningful difference for the problem-solving process, then we can claim local homogeneity. Alternatively, if there is a meaningful difference, this can be incorporated into the model so that different strategies are represented using different attributes, and then we can reclaim local homogeneity (e.g., as in De La Torre and Douglas, 2008; Ma and Guo, 2019). This will allow us to adopt the stochastic interpretation of the item response function and there will be an important theoretical distinction between “true” models and “other” models.

Four challenges can be raised against the claim that “true DCM” can have local homogeneity. First, there is general evidence that the assumption of local homogeneity is invalid. While education tests face the challenge of the test–retest effect (Scharfen et al., 2018), some personality tests do not have this problem because the traits being measured are assumed to be relatively stable and unaffected by the act of taking the test (Borsboom, 2005). Longitudinal studies which have used the same personality tests multiple times with the same individuals have found that patterns of intra-individual differences are distinct from patterns of inter-individual differences (e.g., Borkenau and Ostendorf, 1998; Cervone, 1997). This means the response model for the subpopulation is not the same as the model for the individual. While the structures of personality may not fully match the structures of knowledge and cognition, the results of these studies do warrant caution in adopting the homogeneity assumption.

Secondly, even at the fine-level of analysis in “true” models, there can be strong grounds to challenge the assumption that problem-solving processes are shared by respondents with the same mastery. Certainly, we can expect reasonable homogeneity in the processes of some attributes: it is highly unlikely there are significantly different approaches to *separate whole number from fraction* (Tatsuoka, 1984), for example. However, it is difficult and uncommon for a cognitive model to be composed exclusively of such attributes. For example, there are multiple subtraction techniques that could be employed during *basic fraction subtraction* (counting up, counting down, using number bonds; Tatsuoka, 1984).

A further problem arises from the fact that DCM are ambivalent to the order in which attributes are applied. Figure 3 demonstrates the possibility (however unlikely) that the same set of attributes can be used in different orders to reach a solution. In other cases, such as when there are multiple operators involved, the order in which attributes are applied could make a significant difference to the difficulty of the solution and therefore challenge the idea of local homogeneity.

**Figure 3:**
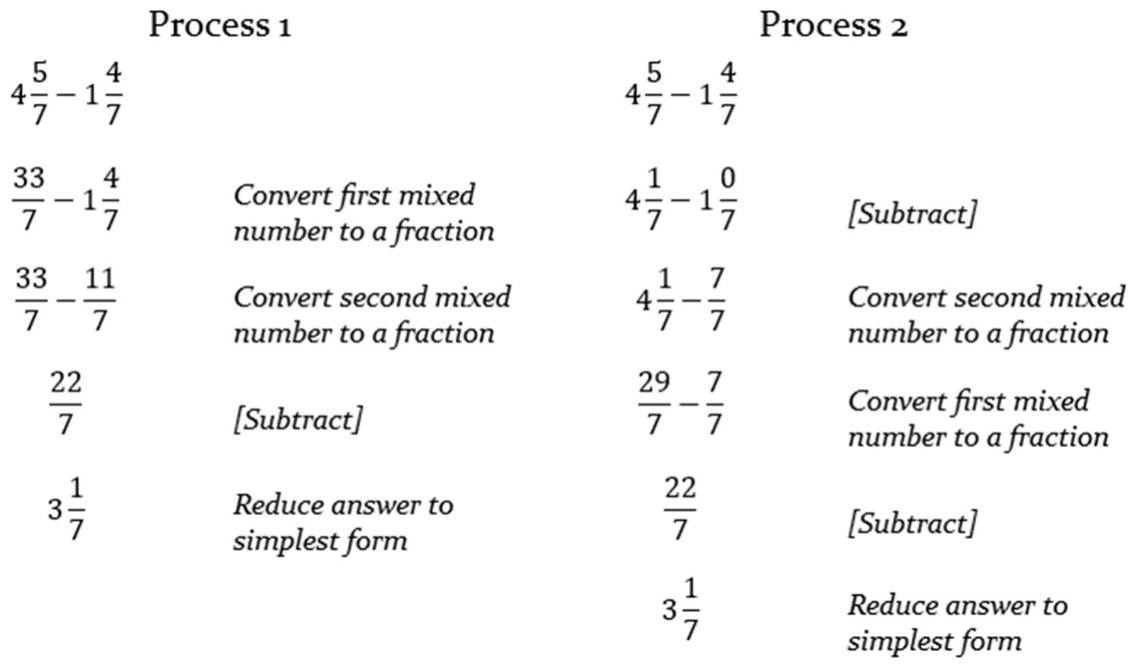
Alternative solution sequences for fraction subtraction.

The final and perhaps most devastating challenge comes from turning attention to non-mastery rather than mastery. Earlier, we had the example that local homogeneity requires that if John, a non-master, were a master like Jane, he would solve the problem following the exact same process as Jane. If we invert this situation, we can also require that if Jane, a master, were a non-master like John, she would fail to solve the problem following the same process as John.

This inversion is possible due to the binary and comprehensive nature of attributes. In a general context, an inversion such as this is not possible. If smoking was the cause of developing lung cancer, we cannot invert this to say the cause of not having lung cancer is not smoking because not having lung cancer is the default state (i.e., uncaused). However, in the case of attributes, an incorrect response does require a cause, since the default state is non-response.

The problem here is that non-masters can differ greatly in their incorrect responses. This ties into the foundations of DCM: in the 1970s and 80s, there was research interest in modelling student mastery and misconceptions simultaneously (e.g., Brown and Burton, 1978). In fact, the paradigmatic fraction subtraction model came from this work, and 74 erroneous rules were identified for adding or subtracting fractions, alongside 11 attributes for solving problems correctly (Tatsuoka, 1984). As this example demonstrates, there are far more ways to be wrong than right. This indicates that even if all masters of an attribute follow the same process to solve a problem, non-masters of the attribute vary in their processes when making errors (Payne, 1990; Sleeman et al., 1989). Moreover, studies found that even individual students were inconsistent in their application of “mal-rules,” applying different misconceptions when solving different problems of the same type. This was a key reason why some concluded focusing only on mastery and non-mastery was more practical, and therefore DCM were promoted over error-focused models (Birenbaum et al., 1992; Birenbaum and Tatsuoka, 1993).

To conclude, the probability of each non-master answering a question correctly can be mathematically derived from the probability of each non-master answering the question incorrectly, and this is determined by their non-mastery. Since the nature of non-mastery is expected to be highly inconsistent, the probability of each individual producing an incorrect response will also be inconsistent (e.g., one student may be randomly guessing while another may consistently apply a misconception). As a result, the item response function can only represent the aggregate probability of non-masters’ producing correct responses rather than the individual probability for each non-master. This means local homogeneity for non-masters does not hold.

As a result of these challenges, the stochastic interpretation of the item response function for “true DCM” is arguably inappropriate, leaving the subpopulation interpretation as the remaining option. This means the grain-size of attributes is not a deciding factor in the interpretation of the item response function, and “true DCM” and “other DCM” are not theoretically distinct in this regard.

## 3 Conclusion

This paper aimed to explore plausible distinctions between attributes in “true DCM” and “other DCM.” It argued that both the fine-grained attributes in “true DCM” and broad attributes in “other DCM” are composites of finer-grained skills but they can both also be treated as singular, so they are not distinct in this regard; it argued that, in the statistical model, both “other” and “true DCM” can assume that attributes represent ontologically real phenomena rather than constructs; and it argued that local homogeneity for “true DCM” is highly unlikely, meaning that both “true” and “other DCM” need to follow the subpopulation interpretation of the item-response function.

The basic conclusion here is that the difference in grain-size of attributes in “true” and “other” cognitive models does not clearly lead to a theoretical boundary. This does not preclude the possibility of other theoretical distinctions, and there may be cases where attributes are simply a collection of disconnected skills or where attributes are considered to be ontological constructs. However, such distinctions should be identified rather than presumed. This paper is, therefore, just one step in establishing whether retrofitting and other DCM studies should operate under a distinct theoretical framework from “true DCM” studies (as is implicit in Gierl and Cui, 2008).

Superficially, this study implies researchers should not be deterred from conducting DCM studies using broad attributes, and poor model fit is not necessarily caused by the grain-size of attributes. However, the arguments in this paper can help explain when broad attributes do yield sub-optimal fit and how this can be avoided.

First, while both fine-grained and broad attributes may be functionally singular, this does not mean they always are. The degree to which an attribute can be considered single depends on how closely sub-skills “hang together” (i.e., the strength of the sustaining mechanism: Boyd, 1991). Fine-grained attributes such as *finding a common denominator* can easily become a cognitive routine through repeated practice. Similarly, although broad skills such as *critical reasoning* and *spoken fluency in a foreign language* emerge from the dynamic interaction of lower sub-skills, they function as singular constructs because mastery of the complex attribute cannot be predicted by sub-skill mastery alone (Liu et al., 2014). In contrast, if executive coordination of sub-skills is limited to identifying task type, this arguably represents an insufficient unifying mechanism. Consequently, attributes such as *measure, estimate, and understand properties of lines and angles* (Lee et al., 2011) might better be modelled as distinct, independent skills rather than as a single cohesive construct, explaining why poor model fit is likely to result from this forced aggregation.

Second, even when attributes are functionally singular, as grain-size increases, partial mastery becomes an increasing issue. In standard testing contexts, an item testing a broad attribute will generally cover a lower proportion of the sub-skills than one testing a fine-grained attribute. To illustrate, a single fraction subtraction question can require many of the sub-skills that compose *finding a common denominator*, whereas a single mathematics question can only target a small portion of the skills that make up *algebra*. In this context, a student who has not mastered *finding a common denominator* but who has mastery of some sub-skills is, nonetheless, unlikely to answer the item correctly, but a student with mastery of the specific sub-skills of *algebra* tested in the item would be likely to answer correctly. When a clear binary classification is used with broad attributes, the responses of individuals can be inconsistent with the model, since they respond reliably as masters to some items and non-masters to others. This results in poor model fit. This is more of a practical challenge than a theoretical distinction, and there are two main routes to addressing it. One solution is to design a broad test to match the broad attribute - this happens with driving tests, for example, which require demonstration of a range of sub-skills to produce a single pass/fail score (and therefore function as a single item). Of course, it can be highly impractical for a test to contain 30 complex, hour-long items. The other solution is the recent development of partial-mastery DCM which classify students on a continuum between 0 and 1, thereby accommodating intermediate levels of proficiency (e.g., Shang et al., 2021).

Finally, although broad attributes may be sufficiently singular, in some assessment contexts they may suffer from being insufficiently distinct. Assessments of horizontal knowledge structures (Bernstein, 2000) – where knowledge is more segmented – are likely to allow for more clearly delineated attributes. In history, for example, *understanding everyday life* in a particular historical period draws on distinct skills compared to *understanding political change*. In contrast, much of *geometry* involves finding unknown values, creating substantial overlap with the domain of *algebra*. This can be an issue because if attributes are not sufficiently distinct, it will be challenging to identify exactly which attribute is tested in an item (i.e., Q-matrix specification) and the overall estimation and diagnostic performance of the DCM will suffer (Xu and Zhang, 2016). As a result, whether broad attributes are sufficiently distinct can be a challenge in certain contexts, but this issue is unlikely to trouble fine-grained attributes.

In closing, it is important to recognize that this argument generally follows the realist assumption that the selection of a model should be based on theoretical grounds (Borsboom et al., 2003; Hood, 2013). If we take an instrumentalist approach where models are pragmatic tools to achieve human goals, then practical considerations can take precedence over theoretical ideals. For example, socioeconomic status is a classic formative construct, but it is not uncommon to find it modelled using a reflective model (e.g., Yin and Reynolds, 2024). Under this framework, whether broad and fine-grained attributes are ontologically real or causally distinct is a secondary concern, and approaches to addressing poor model fit are not constrained by a need for theoretical consistency. However, this does not mean the preceding discussion is irrelevant for an instrumentalist because theoretically consistent models are more likely to have practical utility (to take a side in a long and ongoing philosophical debate; e.g. MacCallum et al., 1992; Putnam, 1975).

Overall, this study did not establish that the grain-size of attributes results in a theoretical boundary between “true” and “other” DCM. Nonetheless, practical challenges such as poor model fit can be far more common when using broad attributes. When faced with these issues, researchers may review whether broad attributes used do have sufficient sustaining mechanism to be functionally singular and whether the attributes may reasonably be considered ontologically real rather than being constructs. If the attributes satisfy these conditions, pursuing technical solutions within the cognitive diagnostic assessment framework may be justified.

## Footnotes

1 Operationalism is a third option (Bridgman, 1927), but there are a number of strong arguments against it (e.g., as found in Bickhard, 2001; Borsboom et al., 2003; Byerly and Lazara, 1973; Chang, 2017; Vessonen, 2021).
